# Using machine learning to explore the characteristics of eye movement patterns and relationship with cognition ability of Chinese children aged 1–6 years

**DOI:** 10.3389/fnhum.2023.1220178

**Published:** 2023-11-23

**Authors:** Shuqing Zhou, Li Hou, Na Wang, Fulin Liu, Ning Wei, Xia Chi, Dongchuan Yu, Xin Zhang, Meiling Tong

**Affiliations:** ^1^Department of Child Health Care, Nanjing Maternity and Child Health Care Hospital, Women’s Hospital of Nanjing Medical University, Nanjing, Jiangsu, China; ^2^School of Pediatrics, Nanjing Medical University, Nanjing, Jiangsu, China; ^3^Key Laboratory of Child Development and Learning Science of Ministry of Education, School of Biological Science and Medical Engineering, Southeast University, Nanjing, China

**Keywords:** eye movement, children, gaze pattern, cognition, machine learning

## Abstract

Researchers have begun to investigate the relationship between eye movement characteristics of gaze patterns and cognitive abilities, and have attempted to use eye-tracking technology as a new method to evaluate cognitive abilities. Traditional eye movement analysis methods typically separate spatial and temporal information of eye movements, mostly analyze averaged data, and consider individual differences as noise. In addition, current eye movement studies on gaze patterns mostly involve adults, while research on infants and toddlers is limited with small sample sizes and narrow age ranges. It is still unknown whether the conclusions drawn from adult-based research can be applied to children. Consequently, eye movement research on gaze patterns in children is necessary. To address the concerns stated above, this study used the Hidden Markov machine learning method to model gaze patterns of 330 children aged 1–6 years while observing faces freely, and analyzed characteristics of eye movement gaze patterns. Additionally, we analyzed the correlation between gaze patterns of 31 toddlers aged 1–3 years and 37 preschoolers aged 4–6 years, and the different dimensions of cognitive abilities. The findings indicated that children exhibited holistic and analytic gaze patterns while observing different faces freely. More children adopted a holistic gaze pattern, and there were age-specific gaze pattern characteristics and regularities. Gaze patterns of toddlers may be correlated with their adaptive abilities and gaze patterns of preschoolers may be correlated with their visual space abilities. Specifically, toddlers aged 1–3 years showed a moderate negative correlation between the H-A scale and the adaptive dimension, while preschoolers aged 4–6 years showed a low negative correlation between the H-A scale and the visual space dimension. This study may provide new insights into the characteristics of children’s eye-movement gaze patterns during face observation, and potentially offer objective evidence for future research aimed at promoting the use of eye-tracking technology in the assessment of toddlers’ adaptive abilities and preschoolers’ visual space abilities in the field of face perception.

## 1 Introduction

Face perception (Ward and Bernier, 2021) is a fundamental cognitive process that involved in the processing of faces, which allows children to obtain important social information such as gender, age, emotion, and race, facilitating their social communication and adaptation to their environment. Eye tracking techniques (Gredebäck et al., 2010; Carter and Luke, 2020), owing to their advantages of objective quantification, non-invasiveness, and ease of operation, have emerged as a promising tool to characterize children’s face perception. Given that children’s brains are still undergoing development, it remains unclear whether the findings derived from adults are generalizable to children.

Gaze patterns, belonging to the category of facial processing mechanisms, are an important branch of face perception research. Researchers from various fields have actively explored the gaze patterns in the face perception process for decades (Mertens et al., 1993; Walker-Smith et al., 2013; Johnson et al., 2015). However, current eye movement analysis methods for gaze patterns primarily rely on analyzing average data, and individual differences are treated as noise. Nevertheless, studies have demonstrated that eye movement behavior exhibits significant individual differences during cognitive tasks (Kelly et al., 2011; Peterson and Eckstein, 2013; Kanan et al., 2015). Hence, appropriate eye movement analysis methods are necessary to reflect individual differences. Furthermore, most eye movement analysis methods cannot reflect eye movement temporal-spatial features (Henderson et al., 2005; Caldara and Miellet, 2011; Wang et al., 2020). With advancements in computer algorithms, machine learning provides a new and powerful method for eye movement research on gaze patterns (Lim et al., 2022).

In eye movement tasks, the current fixation location of eyes is dependent on the preceding fixation location, making eye movement a time series. Hidden Markov Model (HMM) is a statistical model for analyzing time series in machine learning, thus eye movement behavior in visual tasks can be regarded as an HMM random process (Chuk et al., 2014). Studies have indicated that HMM is appropriate for cognitive psychology research involving limited trials or time-intensive data collection, providing a new opportunity for addressing individual differences and spatial -temporal issues in eye movement data analysis (Chuk et al., 2020). Using the HMM method can provide a better understanding of the eye movement process (Hsiao et al., 2021). Previous research (Chuk et al., 2014, 2020; Hsiao et al., 2021) has shown that, according to the transition differences among fixation locations and spatial distribution differences, adults exhibit two representative gaze patterns when gazing at faces: the holistic gaze pattern, characterized by fixation on the midline of the face, and the analytic gaze pattern, which focuses more on the area between the eyes and mouth. However, it is currently unclear how many representative gaze patterns exist in children and what specific characteristics each pattern possesses. Therefore, it is necessary to conduct eye-tracking studies to explore gaze patterns in children.

Researchers have begun to explore the relationship between eye movement characteristics and cognitive abilities, and have attempted to use eye tracking technology as an effective method for assessing cognitive abilities in recent years (Hayes and Petrov, 2016; Hayes and Henderson, 2017; Laurence et al., 2018). In a study involving 34 young adults and 34 older adults (Chan et al., 2018), the relationship between eye movement patterns during face recognition and cognition was analyzed using the HMM. Participants who exhibited an analytic gaze pattern performed better in cognitive tasks regardless of age differences, compared to those who exhibited a holistic gaze pattern. However, it remains unclear whether eye movement patterns during face perception in children are associated with cognitive abilities. Given the plasticity of children’s brains and cognitive abilities during childhood (Kolb et al., 2017), studying the relationship between cognitive abilities and gaze patterns in children can help to understand the developmental patterns and mechanisms in children, providing objective evidence for eye tracking technology as a new method for assessing cognitive abilities in children.

Taken together, to investigate the characteristics of children’s eye-movement gaze patterns, we recruited 816 Chinese children aged 1–6 years to participate in an eye-tracking face perception task and their gaze patterns were modeled by HMM. This is the first time, as far as we are aware, that eye movement characteristics of gaze patterns in children aged 1–6 have been reported. Additionally, we recruited 50 children aged 1–3 and 45 children 4–6 to participate in eye-tracking tasks and cognitive assessments to explore the relationship between gaze patterns and cognition.

## 2 Materials and methods

### 2.1 Experiment 1

#### 2.1.1 Participants

The minimum number of participants required for this study was computed using G*Power 3.1 (Faul et al., 2009), assuming an alpha level of 0.05, a power of 0.90, a medium effect size of 0.25, and 8 groups to be tested using the *F*-test, resulting in a required minimum of 338 participants. The final targeted sample size of 796 was calculated, taking into account a 15% rate of loss to follow-up and approximately 50% of the total data set meeting the inclusion criteria. A total of 816 children (423 boys) aged 1–6 years were recruited for this study from June 2020 to June 2022. The inclusion criteria were as follows: A. birth gestational week ≥37 weeks and birth weight ≥2500 g; B. age 1–6 years; C. passing the physical vision screening and visual development (including corrected visual acuity) was at the same age developmental level; D. no serious physical or neurologic disorders occurred after birth; E. no neurodevelopmental disorders. All children were physically evaluated by two pediatricians. Ultimately, 330 children (158 boys) were analyzed due to 486 exclusions resulting from failure to complete the eye movement task or poor eye movement data quality (see the eye movement data analysis section for details).

#### 2.1.2 Materials

**FIGURE 1:**
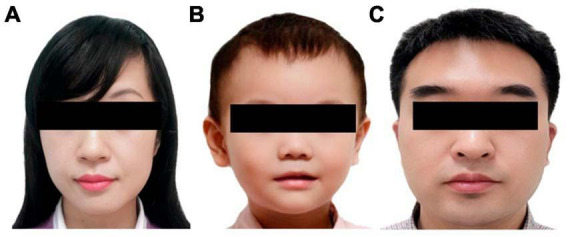
Sample faces as stimuli: **(A)** a female face **(B)** a child’s face **(C)** a male face.

In this experiment, we used three static photos of Chinese faces depicting a female, child, and male as stimuli (Figure 1). All images were standardized to the same shape and size (width: 900 pixels, 18.8° view, height 940 pixels, 22.4° view). Each face image was presented with a neutral expression and oriented in a forward-looking position in a random order. Eye movement was tracked using the Tobii Eye Tracking IS4 Large Peripheral Eye Tracker, with a sampling rate of 90 Hz, and data recording was controlled through the use of the Psychtoolbox and Tobii Analytics Software Development Kit on the Matlab platform. Binocular tracking was used to detect eye movement, with the screen resolution set to 1920 px × 1080 px.

#### 2.1.3 Procedure

The distance between the children and the LCD monitor was about 75 cm. The toddlers were positioned on their parent’s laps, with their parents wearing eye shields to prevent any visual interference, while preschoolers were seated individually in front of the eye tracker on designated chairs. Before each trial, an attention-getter, in the form of a “+,” was presented at the center of the monitor to reduce involuntary movements that may occur, such as tremors, drift, and microsaccades, and to enhance stable fixation (Thaler et al., 2013). Subsequently, each face was shown at the center of the monitor for 8,000 ms, and children were instructed to observe them at will. Children were blinded to the results of the eye tracking assessment.

Before data collection, the children’s eye movements were calibrated through two three-point calibration procedures. During calibration, a blue dot with a 1-cm diameter was used, and the children were instructed to maintain fixation on the dot until it burst and disappeared. The calibration process was repeated when necessary to guarantee good mapping was achieved at all six test positions, each with a visual angle of less than 1°.

### 2.2 Experiment 2

We conducted Experiment 2 to investigate whether the representative gaze models derived from Experiment 1 could potentially be used to quantify the eye-movement patterns of new child participants for cognitive screening. The minimum number of participants required for Experiment 2 was computed using G*Power 3.1, assuming an alpha level of 0.05, a power of 0.80, a medium effect size of 0.32, and 2 groups to be tested using the *t*-test, resulting in a required minimum of 62 participants. The final targeted sample size of 91 was calculated, taking into account a 15% rate of loss to follow-up and approximately 80% of the total data set meeting the inclusion criteria. A total of 95 children (50 toddlers aged 1–3 years and 45 preschoolers aged 4–6 years) were recruited for Experiment 2 from June 2020 to June 2022 (see section “2.1.1. Participants” for inclusion criteria). After performing the same eye movement task, each child completed developmental and behavioral assessments. The developmental assessments included the Gesell Developmental Diagnostic Schedule (GDDS) and the Wechsler Preschool and Primary Scale of Intelligence, Fourth Edition (WPPSI-IV), used for the toddler and preschooler groups, respectively, to examine the relationship between eye movement gaze patterns and various dimensions of cognitive ability. In the toddler group, 31 children (mean age, 25.3 months; SD, 8.3 months, 16 boys) were included in the final analysis, and a total of 37 participants (mean age, 49.20 months; SD, 8.40 months, 16 boys) from the preschooler group underwent the final analysis.

1. Gesell Developmental Diagnostic Schedule (GDDS) (Ball, 1977) evaluates the level of development of a child’s abilities in different systems, including four functional areas: adaptive, motor (gross and fine motor), verbal, and social areas. The results are expressed in terms of the developmental quotient (DQ).

(1) Adaptive: Adaptive behavior is the most important area that reflects the overall development status of children. It involves the organization of stimuli, perception of their relationships, decomposing stimuli into their components, and reassembling these components in a meaningful way. Adaptive behavior is the precursor to future “cognition” as it allows children to apply past experiences to solve new problems.

(2) Motor: Gross motor behavior encompasses postural reactions, head control, sitting, standing, crawling, walking, and more. Fine motor behavior involves actions such as grasping, gripping, and manipulating objects using the hands and fingers.

(3) Verbal: Language behavior includes imitation and comprehension of others’ language.

(4) Social: Personal-social behavior includes infants’ individual responses to the social and cultural context they live in. The response to society and the environment encompasses children’s abilities, attitudes, their eating abilities, independence in play, cooperation, and their responses to training and social customs.

2. Wechsler Preschool and Primary Scale of Intelligence, Fourth Edition (WPPSI-IV) (Wechsler, 2012) includes total scale scores and five subscales—verbal comprehension, visual space, perceptual reasoning, working memory, and processing speed.

(1) Verbal comprehension refers to the ability of subjects to accurately summarize, understand, and express language information through common sense and scores obtained from analogical reasoning tests.

(2) Visual space refers to the subjects’ ability to analyze and organize pattern materials, spatial perception, and visual-motor integration skills, as measured by their scores on block assembly and puzzle tests.

(3) Perceptual reasoning encompasses the subjects’ higher-level thinking abilities, including abstract generalization and reasoning based on picture materials, which are reflected in their scores on matrix reasoning and picture concepts tests.

(4) Working Memory refers to the subjects’ short-term memory abilities for pattern materials, as indicated by their scores on picture memory and animal house tests.

(5) Processing Speed refers to the subjects’ ability to quickly scan and distinguish visual patterns, physically marking them, as determined by their scores on bug search and cancelation tests.

### 2.3 Analysis of eye-movement data

#### 2.3.1 Preprocessing

Trials with more than 50% missing gaze data (a total screen time of less than 4000 ms) were considered unreliable and thus were excluded from the analysis. Missing gaze data in the other trials were filled in by linear interpolation, with a maximum gap length of 75 ms, which was regarded as an eye blink (Olsen, 2012). The average gaze position of the left and right eyes was used as an analytical unit. Regions of interests (ROIs) were not predefined in this research. We computed the total screen time by summing up all gaze durations on the entire screen.

#### 2.3.2 Hidden Markov model

We used the EMHMM method to quantitatively measure eye-movement patterns in a child (Chuk et al., 2014, 2020; Hsiao et al., 2021). In the EMHMM approach, the eye-movement pattern of each child is summarized using the HMM based on personalized ROIs and transition probabilities among ROIs, given that the hidden states of the HMM correspond to these ROIs. The parameters of HMMs, including the Gaussian ROIs, the transition matrix, and the vector of priors (which indicate the probabilities that a fixation sequence starts from the ellipses), were simultaneously estimated using the Variational Bayesian Expectation Maximization algorithm, with the number of ROIs automatically determined from a preset range via the variational Bayesian approach. The HMMs of all children could then be clustered into subgroups based on their similarities by using Variational Hierarchical Expectation Maximization (VHEM), and a representative HMM model could be produced for each subgroup. The eye-movement pattern of each child could be quantitatively evaluated to determine the likelihood of the generated representative HMMs: the higher the likelihood, the more similar the representative HMMs.

In this study, we aimed to determine the optimal number of ROIs for each participant by training six HMMs, with ROIs ranging in number from 1 to 6. The model with the highest log-likelihood was selected. To ensure robustness of the results, each HMM was trained 100 times with unique initial Gaussian ROIs, which prevented convergence to local maximum. Subsequently, we generated a holistic and analytic HMM model representative of the data. To assess the similarity of the eye movement pattern between the child and the representative models, we calculated the H-A scale for each child by subtracting the log-likelihood of the child’s eye movement data from each of the two models, and normalizing the result by its sum (Chan et al., 2018):

$$H-A\,s\,c\,a\,l\,e=\frac{H\,o\,l\,i\,s\,t\,i\,c\,l\,o\,g-l\,i\,k\,e\,l\,i\,h\,o\,o\,d-A\,n\,a\,l\,y\,t\,i\,c\,l\,o\,g-l\,i\,k\,e\,l\,i\,h\,o\,o\,d}{\left|H\,o\,l\,i\,s\,t\,i\,c\,l\,o\,g-l\,i\,k\,e\,l\,i\,h\,o\,o\,d\right|+\left|A\,n\,a\,l\,y\,t\,i\,c\,l\,o\,g-l\,i\,k\,e\,l\,i\,h\,o\,o\,d\right|}$$

**FIGURE 2:**
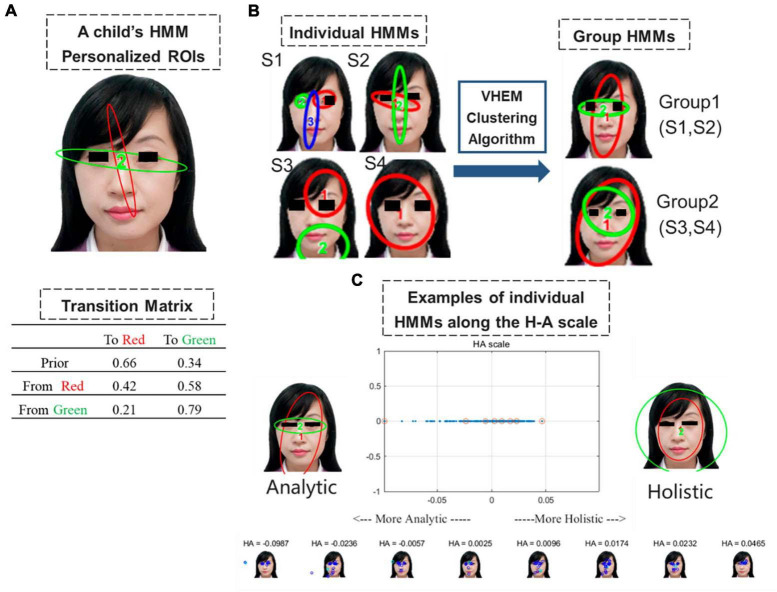
**(A)** Example of an HMM summarizing the eye movement pattern of a child during a free-viewing task. Ellipses depict the ROIs as 2D Gaussian distributions. The table shows transition probabilities among the ROIs. Priors show the probabilities that a fixation sequence starts from the ellipse. In this example, the child has a 66% probability of viewing the female face with fixation in the red region and 34% with fixation in the green region. After fixation in the red region, the subsequent fixation has a 42% probability to stay in the red region and 58% to switch to the green region. **(B)** Illustration of the Variational Hierarchical Expectation Maximization clustering algorithm: The clustering algorithm groups S1 and S2 form Group 1, and S3 and S4 form Group 2. Group 1 and Group 2 HMMs can then be used to quantify the eye movement pattern and derive from it the data likelihood of the child given Group 1 HMM or Group 2 HMM. **(C)** Example of the generated plot showing the analytic and holistic representative models (top-left and top-right) and the H-A scale (top-center). A small number of children on the scale are plotted on the bottom. These corresponding values are highlighted on the H-A scale plot with red circles. Positive HA values indicate a more holistic pattern, whereas negative HA values indicate a more analytic pattern.

A positive value indicates a holistic pattern, whereas a negative value indicates an analytic pattern. We then examined the correlation between the H-A scale and developmental assessments in Experiment 2. The schematic diagram of the HMM is presented in Figure 2.

## 3 Results

### 3.1 Experiment 1

Table 1 presents the basic profiles of children in each age group before and after data cleaning in Experiment 1. Ultimately, 330 children (158 boys and 172 girls, mean age: 46.40 months; SD: 19.59 months) were included in the analysis.

**TABLE 1:** General profile of children in Experiment 1 before and after data cleaning, including the number of children and the age of children in each age group.

	Before data cleaning		After data cleaning	
	N (M/F)	Age (months), mean, SD	N (M/F)	Age (months), mean, SD
1Y, 1.5Y)	152 (77/75)	12.67 (2.05)	29 (12/17)	12.89 (2.32)
1.5Y, 2Y)	70 (30/40)	19.67 (2.23)	23 (8/15)	19.96 (2.48)
2Y, 2.5Y)	74 (35/39)	25.44 (1.82)	21 (10/11)	25.34 (2.03)
2.5Y, 3Y)	77 (44/33)	31.21 (1.74)	32 (16/16)	30.71 (1.99)
3Y, 4Y)	140 (69/71)	40.80 (4.00)	50 (23/27)	41.41 (4.19)
4Y, 5Y)	141 (85/56)	53.45 (3.88)	73 (43/30)	53.02 (3.61)
5Y, 6Y)	121 (65/56)	66.06 (3.57)	70 (34/36)	66.50 (3.46)
6Y, 7Y)	41 (18/23)	73.97 (2.53)	32 (12/20)	73.96 (2.39)

N, number; M, male; F, female; SD, standard deviation.

**FIGURE 3:**
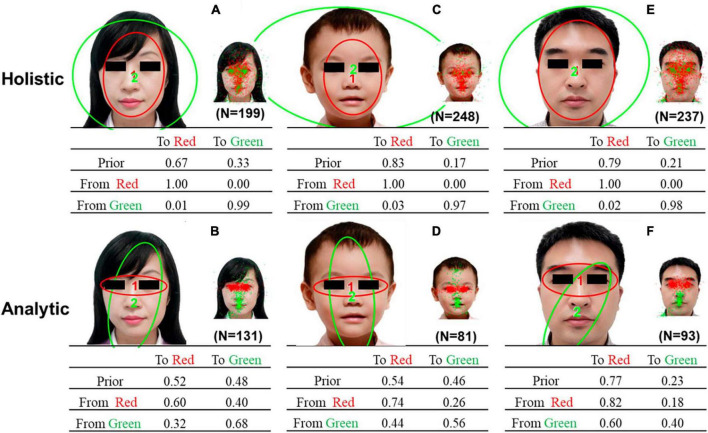
Representative HMMs discovered via clustering by HMM similarities. **(A)** holistic pattern of the female face, **(B)** analytic pattern of the female face, and **(C)** holistic pattern of the child’s face, **(D)** analytic pattern of the child’s face, **(E)** holistic pattern of the male face, and **(F)** analytic pattern of the male face. Each representative HMM included two regions of interest (ROIs) indicated by different colors. The smaller images show the assignment of actual fixations to different ROIs. The transition probabilities of eye movements among the ROIs were summarized in the transition matrix.

Figure 3 presents the representative HMMs of the two common patterns discovered via clustering by HMM similarities. The patterns in Figures 3A, C, E resemble the holistic patterns: a scan path typically starts at the facial contour (the area between the eyes, nose, and mouth) or the entire face and surrounding area, and then lingering around the same area. The patterns in Figures 3B, D, F resemble analytic patterns: a scan path is typically switched between the eyes and the center of the face, with more frequent gazing between the eyes.

Table 2 compares the differences between two representative HMMs using Kullback-Leibler divergence estimation. The results indicate that there are significant differences between the two representative HMMs. Additionally, a chi-square test revealed that a larger number of children exhibited holistic gaze patterns when gazing at a female face (χ^2^ = 14.01, *P* < 0.001), a child’s face (χ^2^ = 85.53, *P* < 0.001), and a male face (χ^2^ = 62.84, *P* < 0.001).

**TABLE 2:** Compares the difference between two representative HMMs.

		Holistic	Analytic	χ2	*P*
Female face	N	199	131	14.01	<0.001
	*d*	0.23	2.46		
	*t*	3.31	28.12		
	*P*	<0.001	<0.001		
Child face	N	248	81	85.53	<0.001
	*d*	0.10	2.31		
	*t*	1.51	20.82		
	*P*	<0.001	<0.001		
Male face	N	237	93	62.84	<0.001
	*d*	0.58	2.71		
	*t*	8.98	26.14		
	*P*	<0.001	<0.001		

The variable ‘d’ in the table represents the Kullback-Leibler (KL) divergence, which is a measure of the difference between probability distributions. A KL divergence of 0 means that the two distributions (HMMs) are the same. The t-statistic in the table is calculated by conducting a paired t-test on two lists of log-likelihoods to determine whether the average log-likelihood difference is significantly different from 0. The χ^2^ statistic at the right of the table is calculated based on a chi-square test conducted to compare the number of participants with holistic and analytical patterns. N, number; d, KL divergence.

**FIGURE 4:**
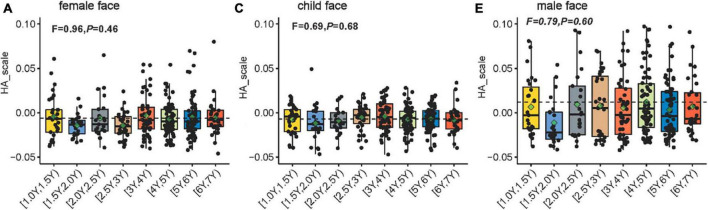
Scatterplots showing the H-A scale for each age group of 330 children aged 1–6 years free-viewing **(A)** a female face, **(B)** a child’s face, and **(C)** a male face. The H-A scale is defined as the difference in the log-likelihoods of the eye movement data of the child (generated using the representative holistic and analytic HMM models), divided by the sum of the two log-likelihoods. Dashed lines depict the average of the H-A scale for all children. The green rhombus indicates the average H-A scale for each age group. The boxplots show the median, range, and first/third quartiles. The notch ranges indicate 95% confidence intervals around the median. The *F* statistic was obtained using one-way ANOVA conducted to compare the H-A scale across age groups.

Figure 4 shows the scatterplots and boxplots of the H-A scale for each age group of children free-viewing a female face, a child’s face, and a male face. The one-way ANOVA on the H-A scale of viewing different faces indicates no significant difference in the H-A scale among age groups (female face, *F* = 0.96, *P* = 0.46; child’s face, *F* = 0.69, *P* = 0.68; male face, *F* = 0.79, *P* = 0.60). To understand how face type and age affect eye movement patterns in children (H-A scale), we performed an 8 × 3 between-subjects ANOVA on the H-A scale (i.e., 8 age groups [1Y, 1.5Y), [1.5Y, 2Y), [2Y, 3Y), [2.5Y, 3Y), [3Y, 4Y), [4Y, 5Y), [5Y, 6Y), [6Y, 7Y) groups) × 3 (face type: female, child, male). The results showed the main effect of face types on the H-A scale (*F* = 42.07, *P* < 0.01). Meanwhile, the age groups exerted no significant effect on the H-A scale (*F* = 1.44, *P* = 0.19). No significant interaction was found between face types and age groups (*F* = 0.50, *P* = 0.93). This result indicates that the H-A scale of different faces varied in essentially the same pattern and that each scale was at its own level as the age group level changed.

**FIGURE 5:**
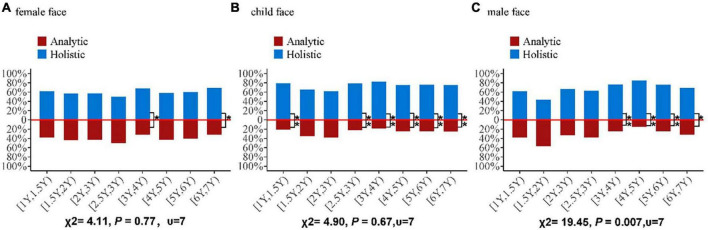
Bar charts showing the proportions of eye movement patterns (analytic and holistic) for each age group of children (aged 1–6 years) free-viewing **(A)** a female face, **(B)** a child’s face, and **(C)** a male face. The proportion of the eye-movement pattern is determined by the number of children adopting the holistic pattern (or analytic pattern) for each group, divided by the total number of children belonging to that particular age group. The blue bar indicates the holistic proportion; the red bar indicates the analytic proportion. The χ^2^ statistic at the bottom of the figure is calculated based on a chi-square test conducted to compare the proportions of holistic and analytical patterns across age groups. *At the top of the bar indicates the type of chi-square test performed to compare the proportions of holistic and analytic patterns in each age group (*indicates *P* < 0.05, **indicates *P* < 0.01).

One of the questions put forward is whether the eye movement pattern would change with age. To address this concern, we explored the proportion of eye movement patterns adopted by the children when viewing different faces. The bar charts in Figure 5 represent the proportions of the eye-movement patterns (analytic and holistic) adopted by children (aged 1–6 years) free-viewing different faces. We conducted a chi-square test on the distribution of the eye-movement patterns adopted by children viewing different faces. No significant differences in the distribution of eye movement patterns were found among the age groups for female faces and child’s faces (female face: χ^2^ = 4.11, *P* = 0.77; child’s face: χ^2^ = 4.90, *P* = 0.67). While the distribution of eye movement pattern proportion was significantly different among the age groups for male faces (male face: χ^2^ = 19.45, *P* < 0.01). In addition, we performed chi-square tests on the distribution of the eye-movement patterns (holistic and analytic) adopted by the children viewing different faces. Significant differences in the distribution of eye-movement patterns when viewing female faces were found in the [3Y, 4Y) and [6Y, 7Y) groups ([3Y, 4Y): χ^2^ = 6.48, *P* = 0.01; [6Y, 7Y): χ^2^ = 4.50, *P* = 0.03). Similarly, significant differences in the distribution of eye-movement patterns during free-viewing of the child’s face were found in the six age groups, except for the [1.5Y, 2Y) and [2Y, 2.5Y) groups ([1Y, 1.5Y): χ^2^ = 9.97, *P* < 0.01; [2.5Y, 3Y): χ^2^ = 10.13, *P* < 0.01; [3Y, 4Y): χ^2^ = 20.48, *P* < 0.01; [4Y, 5Y): χ^2^ = 18.75, *P* < 0.01; [5Y, 6Y): χ^2^ = (18.51, *P* < 0.01; [6Y, 7Y): χ^2^ = 8.00, *P* < 0.01). Significant difference was indicated in the distribution of eye-movement patterns in the [3Y, 4Y) to [6Y, 7Y) groups viewing the male face ([3Y, 4Y): χ^2^ = 13.52, *P* < 0.01; [4Y, 5Y): χ^2^ = 35.63, *P* < 0.01; [5Y, 6Y): χ^2^ = 18.51, *P* < 0.01; 6-year-old group: χ^2^ = 4.50, *P* = 0.03). To understand how face types and age groups affect the eye movement patterns of the children, we performed an 8 age groups × 3 face types between-subjects ANOVA (8 age groups: [1Y, 1.5Y), [1.5Y, 2Y), [2Y, 2.5Y), [2.5Y, 3Y), [3Y, 4Y), [4Y, 5Y), [5Y, 6Y), [6Y, 7Y)) × 3 (face types: female, child, male) between-subjects ANOVA. The results showed that face type and age group were the main factors affecting eye movement patterns (face types: *F* = 15.16, *P* < 0.01; age groups: *F* = 3.01, *P* < 0.01). No significant interaction was identified between face type and age group (*F* = 1.55, *P* = 0.08). This result suggests that the change in eye-movement patterns is similar for different faces as the age group advances, and each is on its own trajectory.

### 3.2 Experiment 2

In the toddler group, 31 children (mean age, 25.3 months; SD, 8.3 months, 16 boys) were included in the final analysis, and a total of 37 participants (mean age, 49.20 months; SD, 8.40 months, 16 boys) from the preschooler group underwent the final analysis. Table 3 presents descriptive statistics of eye movement hidden Markov models (HMMs) and developmental assessments after data cleaning in Experiment 2.

**TABLE 3:** Summary of the clinical characteristics of children in Experiment 2.

	Mean (SD) toddler (*N* = 31)	Mean (SD) preschooler (*N* = 37)
M/F	16/15	16/21
Age, months	25.3 (8.3)	49.2 (8.4)
**Eye movement patterns (N)**		
Female face (H/A)	26/5	30/7
Child’s face (H/A)	19/12	13/24
Male face (H/A)	13/18	18/19
**H-A scale**		
Female face	−0.0175 (0.0342)	0.0133 (0.0399)
Child’s face	0.0145 (0.0242)	0.0019 (0.0275)
Male face	−0.0031 (0.0286)	−0.0078 (0.0248)
Mean face	−0.0020 (0.0162)	0.0025 (0.0184)
**GDDS (DQ)**		
Adaptive	97 (7)	——
Gross motor	89 (8)	——
Fine motor	95 (10)	——
Verbal	95 (13)	——
Social	96 (10)	——
**WPPSI-IV (standard score)**		
Total score	——	113 (11)
Verbal comprehension	——	114 (13)
Visual space	——	110 (12)
Fluid reasoning	——	109 (10)
Working memory	——	107 (9)

Values are presented as means (standard deviations are noted in parentheses unless otherwise noted). M, male; F, female; H, holistic; A, analytic; GDDS, Gesell Developmental Diagnostic Schedule; DQ, developmental quotient; WPPSI-IV, Wechsler Preschool and Primary Scale of Intelligence | Fourth Edition.

**FIGURE 6:**
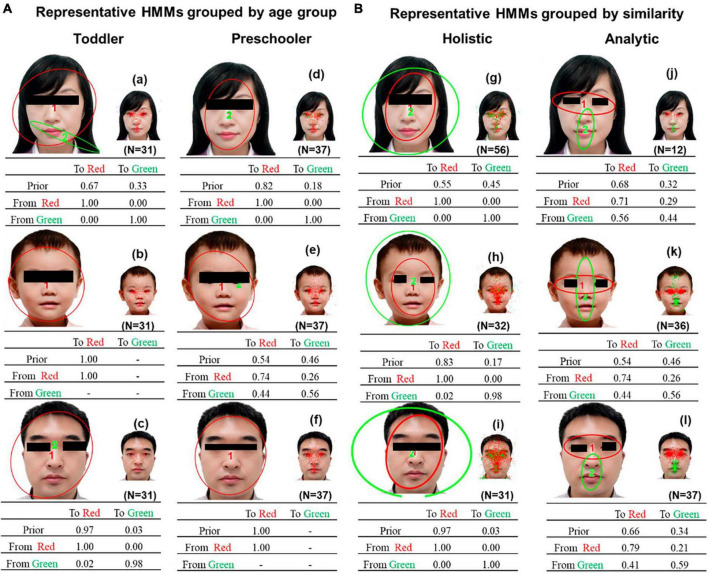
**(A)** Representative HMMs grouped by age group. **(B)** Representative HMMs discovered by clustering based on HMM similarities. Each representative HMM includes one or two regions of interest (ROIs), as indicated by the different colors. Smaller images show the assignment of actual fixations to different ROIs. The transition probabilities of eye movements among the ROIs are summarized in the transition matrix.

Figure 6A shows the representative eye movement patterns obtained by age group. Figure 6B shows the representative HMMs of the two common patterns discovered by clustering based on HMM similarities. The two representative HMMs are significantly different, as determined by Kullback–Leibler divergence estimation (using data from the holistic HMMs of the female face, *t*(55) = 4.13, *P* < 0.001, *d* = 0.55; using data from the analytic HMMs of the female face, *t*(11) = 12.96, *P* < 0.001, *d* = 3.74; using data from the holistic HMMs of the child’s face, *t*(31) = 6.77, *P* < 0.001, *d* = 1.20; using data from the analytic HMMs of the child’s face, *t*(35) = 2.41, *P* = 0.01, *d* = −0.39; using data from the holistic HMMs of the male face, *t*(30) = 3.80, *P* < 0.001, *d* = 0.68; using data from the analytic HMMs of the male face, *t*(36) = 6.62, *P* < 0.001, *d* = 1.09).

**FIGURE 7:**
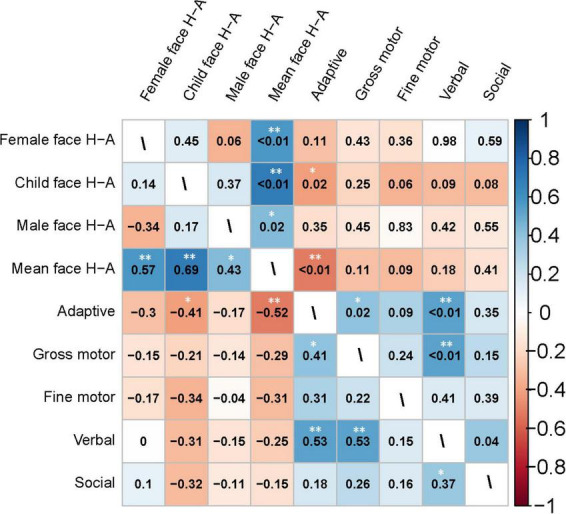
Heatmap of the correlation matrix generated using Spearman’s method for the clinical characteristics of the toddler group. The heatmap of the correlation coefficients is shown in the lower left triangle matrix. *P*-values are in the upper-right triangle matrix. *Significant at *P* < 0.05; **Significant at *P* < 0.01.

**FIGURE 8:**
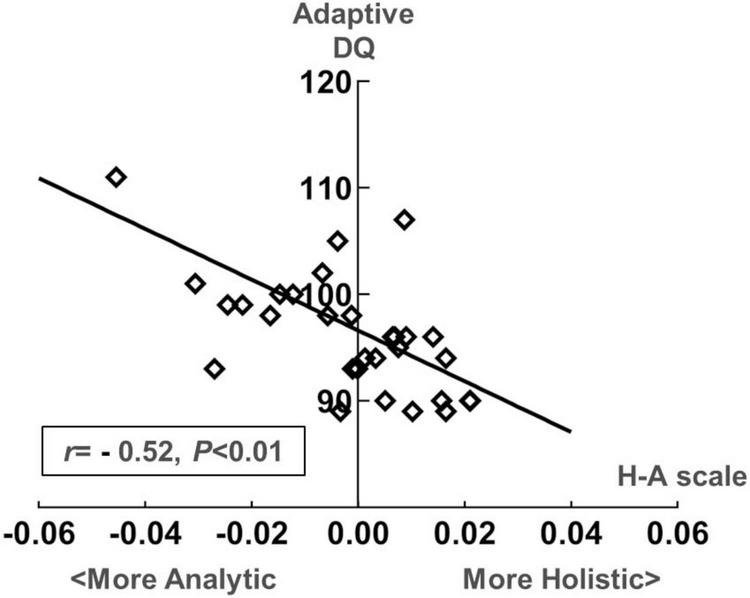
Scatter plot of the development quotient (DQ) in the adaptive dimension of the Gesell Developmental Diagnostic Schedule (GDDS) and the H-A scale in the toddler group.

Figure 7 presents the correlation analysis of the H-A scale with GDDS in the toddler group. The relationship between eye-movement patterns and adaptive dimension (*r* = −0.520, *P* < 0.01) is moderate negative correlation in the toddler group, as shown in Figure 8.

**FIGURE 9:**
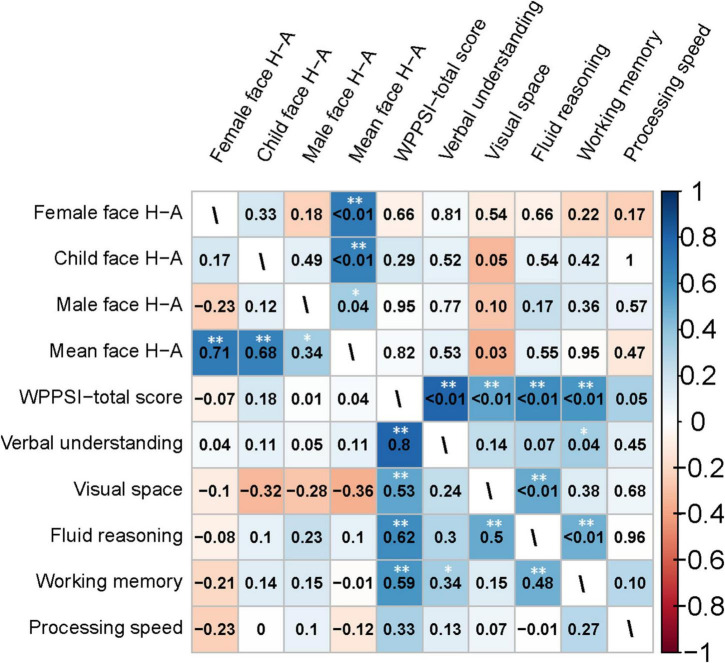
Heatmap of the correlation matrix generated using Spearman’s method for the clinical characteristics of the preschooler group. The heatmap of the correlation coefficients is shown in the lower left triangle matrix. *P*-values are in the upper-right triangle matrix. *Significant at *P* < 0.05; **Significant at *P* < 0.01.

**FIGURE 10:**
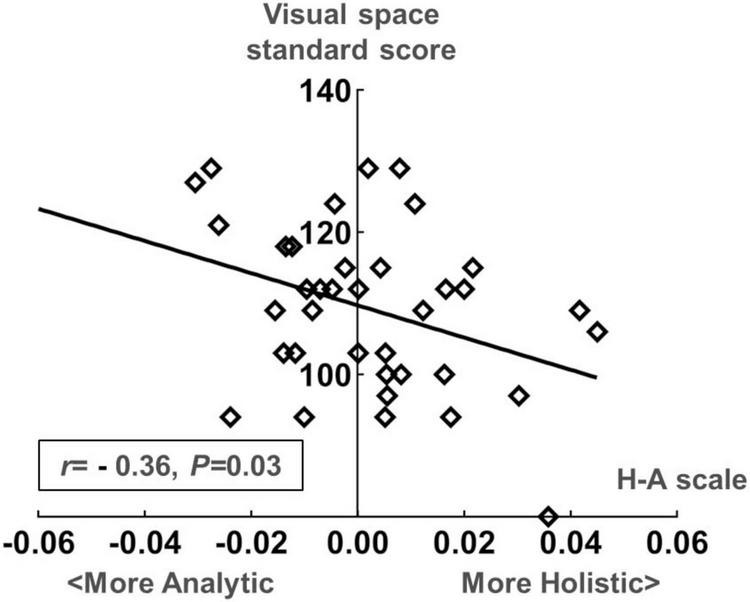
Scatter plot of the standard score in the visual space dimension of the Wechsler Preschool and Primary Scale of Intelligence| Fourth Edition (WPPSI-IV) and the H-A scale in the preschooler group.

We conducted a Spearman correlation analysis between the H-A scale and the standard scores of various dimensions in WPPSI-IV for preschool-aged children. Results showed a significant negative correlation (*r* = −0.36, *P* = 0.03) between the mean values of H-A scale and visual space standard scores, whereas no significant correlation was found (*P* > 0.05) among other indicators. Please see Figures 9, 10 for further details.

## 4 Discussion

### 4.1 Children exhibit two gaze patterns when gazing at faces

In this study, we used an HMM-based approach to investigate eye-movement patterns in children aged 1–6 years during a free-viewing task. The findings revealed two distinct gaze patterns, holistic and analytical, in children while they observed faces (Figures 3, 6B). Notably, these patterns differed significantly. The scan path of the holistic gaze pattern typically starts at the facial contour or the entire face and surrounding area, and then lingering around the same area. While the analytic gaze pattern, typically switches between the eyes and the center of the face, with more looking time toward the eyes (Supplementary Figure 2). Correspondingly, a study focused on eye movement patterns in adults found that two gaze patterns were exhibited by young and older adults when gazing at faces (Chuk et al., 2014). This observation aligns with our study’s outcomes. According to studies on adults, the typical holistic gaze pattern of adults typically starts from the nose or mouth area, then remains near that area. The analytical gaze pattern usually starts from the face’s vertical midline, and then shifts evenly among the midline, left eye, and right eye, with more frequent gaze between the eyes. Each pattern contains three ROIs that partially differed from our study’s conclusions. Our research found that children’s holistic gaze pattern predominantly starts by looking at the whole face, whereas the analytical gaze pattern’s primary focus is both eyes. Each representative gaze pattern has two ROIs. Therefore, our study proposes that despite children and adults having both holistic and analytical gaze patterns, there are dissimilarities between each pattern’s specificities in adults.

We compared the gaze patterns obtained from age-based (Figure 6A) and HMM similarity-based (Figure 6B) methods. Our results revealed that the holistic and analytical gaze patterns obtained from the HMM similarity-based method showed distinct spatial separation within facial features. Since children in both age groups had both holistic and analytical gaze patterns, this difference was not easily observed in the gaze patterns obtained by age-group-based method, which also demonstrated the power of HMM machine learning method.

### 4.2 More children adopt the holistic gaze pattern

Our results indicate that more children exhibit the holistic pattern when gazing at faces (Figure 5), which is a novel discovery as it has not been reported in previous research. In a separate study on eye-movement patterns of adults (Chan et al., 2018), it was found that young adults are prone to adopting the analytical gaze pattern, whereas older adults are more likely to exhibit the holistic gaze pattern while observing faces. Conversely, our research indicates that children tend to employ the holistic gaze pattern, primarily, when viewing faces. Therefore, this may suggest that the facial processing strategies in children aged 1–6 years may still be at the developmental phase and have not fully matured.

### 4.3 Children’s gaze patterns on faces show some characteristics with age

The results of this study show that the ratio distribution of children in each age group using holistic and analytical gaze patterns remains relatively stable with age (Figures 4, 5). Multivariate ANOVA was conducted to understand how face type and age group influence eye-movement patterns in children. On the basis of the results, face type was identified as the main factor affecting eye-movement patterns and the H-A scale, and no significant interaction was found between face type and age group. The results suggest that changes in eye-movement patterns and H-A scale follow essentially the same trajectory for different faces as the age-group advances, and each is at a different level.

### 4.4 Gaze patterns may be correlated with toddlers’ adaptive dimension and preschoolers’ visual space dimension

In Experiment 2, we examined how eye-movement patterns are associated with the subdomains of scales in children aged 1–6 years. To be more specific, toddlers aged 1–3 years showed a moderate negative correlation between the H-A scale and the adaptive dimension (*r* = −0.520, *P* < 0.01) (Figures 7, 8), implying a better adaptive performance associated with the analytical gaze pattern. While preschoolers aged 4–6 years showed a low negative correlation between the H-A scale and the visual space (*r* = −0.360, *P* = 0.03) (Figures 9, 10), indicating a better visual space scores of adopting an analytical gaze pattern. Our findings demonstrate that the eye-movement patterns may be correlated with toddlers’ adaptive dimension and preschoolers’ visual space dimension. As the most important functional domain of GDDS, the adaptive dimension reflects the overall development status of children. It serves as the cornerstone for future cognition development. The moderate negative correlation between H-A values and the adaptive dimension suggests that toddlers who exhibit an analytical gaze pattern may have relatively better scores in the adaptive domain, indicating the potential for better future cognition development. However, prospective research is needed to further investigate this possibility.

### 4.5 Possible mechanisms of face gaze patterns

Based on the recent literature, children who engage in an analytical eye movement pattern may be involved in both local and global processing, whereas those exhibiting a holistic gaze pattern may mainly be involved in global processing (Miellet et al., 2011). The approach that actively retrieves local feature information and global configuration information through an analytic eye movement pattern may lead to optimal face recognition (Bonnen et al., 2012). This is also supported by neuroimaging evidence, as an fMRI study on adults found differences in brain activation between participants who used an analytic versus holistic gaze pattern, with the former showing more activation of brain areas related to face perception (Chan et al., 2017). The results suggest that children who exhibit an analytical gaze pattern may engage more actively in planning eye movements and top-down visual attention (Katsuki and Constantinidis, 2014; Chan et al., 2017) (driven by endogenous factors) during face processing, as indicated by the observed higher transfer of ROIs within analytical gaze patterns. Top-down visual attention has been shown to enhance cognitive task performance by filtering out irrelevant information and selecting useful visual cues (Rutman et al., 2010; Gilbert and Li, 2013). Increased engagement in active eye movement planning and visual attention control while performing the task may explain this performance advantage. In summary, based on indirect evidence from literature, children who exhibit an analytical eye movement pattern may contain local facial feature information (including important facial regions such as the eyes, nose, and mouth) as well as global configuration information (overall facial contour). Specifically, they may employ more efficient strategies, such as actively engaging in eye movement planning (through frequent switching within areas of interest) and top-down visual attention (driven by endogenous factors, filtering out irrelevant information and selecting useful visual cues), to enhance cognitive task performance. However, further mechanistic research is needed to validate the potential.

### 4.6 Practical implications

Eye-tracking technology offers benefits in terms of objectivity, simplicity, speed, and cost-effectiveness (Carter and Luke, 2020). In recent years, researchers from various fields have begun to try using eye-tracking technology as a convenient method for assessing cognitive abilities. Xu et al. (2021) have demonstrated the effectiveness of eye-tracking technology in evaluating cognitive abilities in both normally developing children and those with delayed development. Oyama et al. (2019) have used eye-tracking to quickly assess cognitive function in patients with dementia. In both cases, the cognitive scores obtained from the eye-tracking correlated well with those derived from neuropsychological tests.

The findings from our study indicate a moderate negative correlation between the H-A scale and the adaptive dimension in toddlers aged 1–3 years, as well as a low negative correlation between the H-A scale and visual space in preschoolers aged 4–6 years. This suggests a potential association between toddlers’ gaze patterns and the adaptive dimension. Eye-tracking tasks that incorporate face perception have the potential to be applied to the evaluation of adaptive dimension in toddlers and visual space dimension in preschoolers, and there is potential for their clinical applications. However, additional studies are required to advance this potential.

### 4.7 Limitations and future directions

This study relied on a cross-sectional approach, while a longitudinal cohort design should be used to investigate whether gaze patterns change in individual children during age-related changes in the future. Additionally, due to the COVID-19 pandemic, the recruitment of children from different administrative regions in Nanjing became challenging during the later stages of the experiment. Children participating in Experiment 2 were primarily recruited from our hospital for physical examinations. Future studies should enhance the scientific rigor of sampling by employing techniques like stratified random sampling to ensure a representative sample of the entire population. Thirdly, the study only demonstrates a moderate correlation between toddlers’ eye-movement patterns and adaptive dimensions. Further research is needed in the future to investigate whether these findings hold true for older children. Moreover, expanding the age range of children is also crucial for identifying the point at which children’s cognitive processing of faces transitions from a holistic to an analytic approach. This is essential in studying children’s cognitive gaze patterns of faces. Fourthly, the combined use of technologies such as eye-tracking, fMRI, EEG, and fNIRs, is recommended to explore the potential mechanism of face gaze patterns. Fifthly, despite the moderate correlation found between gaze patterns and the adaptive dimension, as well as the low correlation between children’s gaze patterns and the visual space dimension in this study, there remains a gap in the utilization of eye-tracking technology for assessing children’s adaptive and visual space dimensions. Future studies are needed to promote the potential application of eye-tracking tasks involving face perception in the assessment of children’s adaptive and visual space abilities.

## 5 Conclusion

By using eye-movement data analysis based on HMM, we found that children exhibit two gaze patterns while freely viewing different faces. The first pattern, referred to as the holistic gaze pattern, entails a scan path that typically starts at the facial contour or the entire face and surrounding area, and then lingering around the same area. While the second pattern, known as the analytic gaze pattern, typically switches between the eyes and the center of the face, with a greater tendency to fixate on the eyes. Children are more inclined to manifest the holistic gaze patterns. The gaze patterns of children aged 1–6 exhibit age-related characteristics and regularities. In addition, the eye-movement patterns may be correlated with toddlers’ adaptive dimension and preschoolers’ visual space dimension, and those who exhibit an analytic pattern score higher on these dimensions, implying that eye-movement tasks associated with face perception have potential for assessing toddlers’ adaptive dimension and preschoolers’ visual space dimension in future studies.

## Data availability statement

The raw data supporting the conclusions of this article will be made available by the authors, without undue reservation.

## Ethics statement

The studies involving humans were approved by the Ethical Committee of the Women’s Hospital of Nanjing Medical University (Nanjing Maternity and Child Health Care Hospital). The studies were conducted in accordance with the local legislation and institutional requirements. Written informed consent for participation in this study was provided by the participants’ legal guardians/next of kin. Written informed consent was obtained from the individual(s), and minor(s)’ legal guardian/next of kin, for the publication of any potentially identifiable images or data included in this article.

## Author contributions

MT and XZ developed the initial idea of the study. MT, XZ, and SZ contributed to the study design. FL and DY provided the computer technical support. XC and NiW provided the clinical case support. SZ, LH, and NaW collected the data. SZ and LH did the analyses. SZ and LH wrote the manuscript under the supervision of MT. All authors contributed to the article and approved the submitted version.
