# Supplementary material for: Using machine learning to explore the characteristics of eye movement patterns and relationship with cognition ability of Chinese children aged 1–6 years
Source: Front Hum Neurosci. 2023 Nov 23;17:1220178. doi: 10.3389/fnhum.2023.1220178 (PMC10702211; doi:10.3389/fnhum.2023.1220178)
Supplement: Supplementary file 1 [file Data_Sheet_1.docx]

**Supplementary materials**


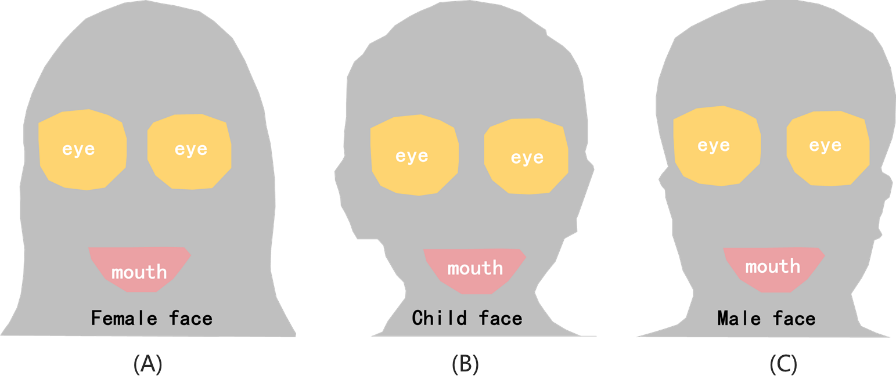


**Figure S1**. Sample ROIs of eyes and mouth (ROIs were not seen by the children during the experiment): (A) a female face (B) a child’s face (C) a male face


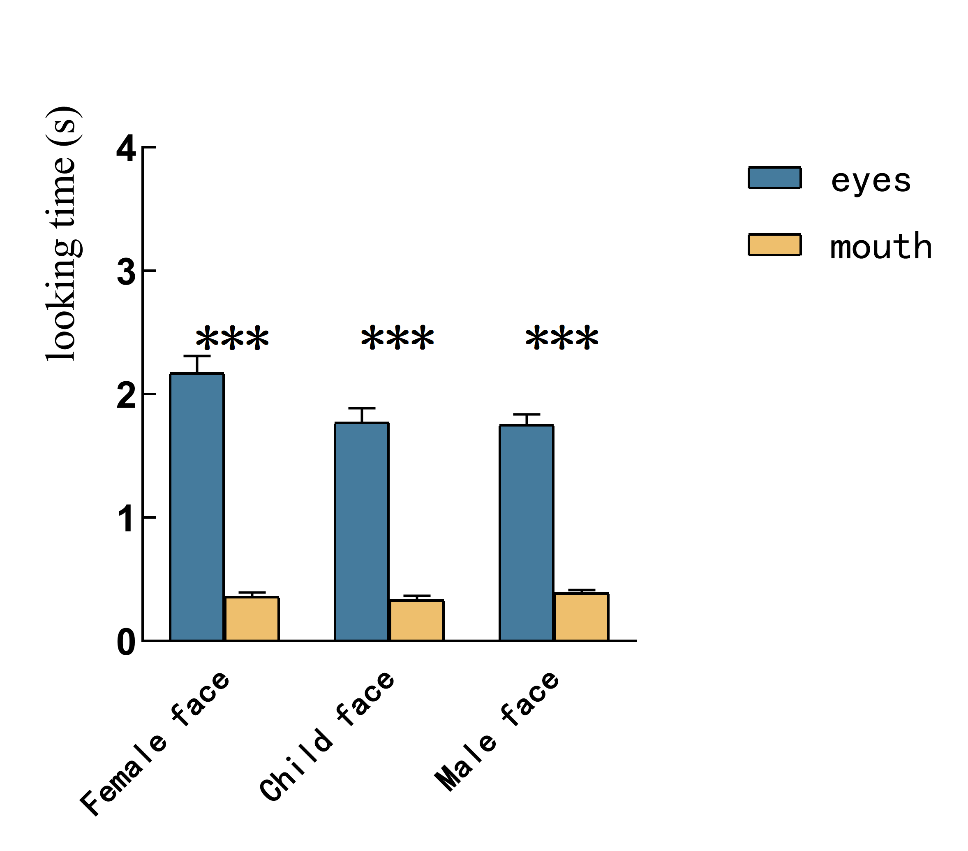


**Figure S2**. Looking time on the eyes and mouth of different faces of the analytic gaze patterns (error bars denote standard errors; *** denotes *P* <0.001).
